# Application of digital-intelligence technology in the processing of Chinese materia medica

**DOI:** 10.3389/fphar.2023.1208055

**Published:** 2023-08-24

**Authors:** Wanlong Zhang, Changhua Zhang, Lan Cao, Fang Liang, Weihua Xie, Liang Tao, Chen Chen, Ming Yang, Lingyun Zhong

**Affiliations:** ^1^ College of Pharmacy, Jiangxi University of Chinese Medicine, Nanchang, Jiangxi, China; ^2^ Nanchang Research Institute, Sun Yat-sen University, Nanchang, Jiangxi, China; ^3^ College of Physical Culture, Yuzhang Normal University, Nanchang, Jiangxi, China; ^4^ School of Biomedical Sciences, University of Queensland, Brisbane, QLD, Australia; ^5^ Key Laboratory of Modern Chinese Medicine Preparation of Ministry of Education, Jiangxi University of Chinese Medicine, Nanchang, Jiangxi, China

**Keywords:** digital and intelligent technologies, Chinese medicine processing, industrialization, standardization, application progress

## Abstract

Processing of Chinese Materia Medica (PCMM) is the concentrated embodiment, which is the core of Chinese unique traditional pharmaceutical technology. The processing includes the preparation steps such as cleansing, cutting and stir-frying, to make certain impacts on the quality and efficacy of Chinese botanical drugs. The rapid development of new computer digital technologies, such as big data analysis, Internet of Things (IoT), blockchain and cloud computing artificial intelligence, has promoted the rapid development of traditional pharmaceutical manufacturing industry with digitalization and intellectualization. In this review, the application of digital intelligence technology in the PCMM was analyzed and discussed, which hopefully promoted the standardization of the process and secured the quality of botanical drugs decoction pieces. Through the intellectualization and the digitization of production, safety and effectiveness of clinical use of traditional Chinese medicine (TCM) decoction pieces were ensured. This review also provided a theoretical basis for further technical upgrading and high-quality development of TCM industry.

## 1 Introduction

Processing of Chinese Materia Medica (PCMM) refers to the processing Chinese botanical drugs by roasting, firing, frying, washing, soaking, bleaching, steaming, boiling and other methods. The purpose of processing is to eliminate or reduce the toxicity of the drug, to enhance the efficacy, to facilitate medicine preparation and storage, and to purify the botanical drugs. Under the guidance of the traditional Chinese medicine (TCM) theory, the proper processing is designed along the therapeutic nature of the drug and eliminates or balances the bias and toxicity, the effects of ascending, descending, floating and sinking, and the meridian tropism of drug, *etc.* So the corresponding changes of drug processing will improve the efficacy and indications, reduce the toxicity, enhance the clinical efficacy, and increase the clinical use. The theory of processing medicine and the theory of differential usage of the raw and cooked medicine were reported (Zhong et al., 2019; Zhai et al., 2020). For example, toxicity of *Aconitum carmichaelii* Debeaux [Ranunculaceae; *Aconite lateralis* radix praeparata] was reduced by heating with hydrolysis of toxic ingredients (Wang et al., 2023). After the dried *Rehmannia glutinosa* (Gaertn.) DC. [Orobanchaceae; *Rehmannia* radix] was made into *Rehmanniae* radix praeparata by multiple steaming and drying with yellow rice wine, its property changed from cold to warm, and its efficacy changed from clearing to tonifying. The steaming and drying repeats created the medicinal components with antioxidant activity of *rehmannia* radix (Kim et al., 2019; Xia et al., 2020; Li et al., 2022). After steaming with black bean juice, the efficacy of *Reynoutria* multiflora (Thunb.) Moldenke [Polygonaceae; *Polygoni multiflora* radix] was changed from diarrhea to tonifying (Gu et al., 2022; Song et al., 2022). The analgesic effect was enhanced by vinegar-processing *Corydalis* yanhusuo (Y.H.Chou and Chun C.Hsu) W.T.Wang ex Z.Y.Su and C.Y.Wu [Papaveraceae; *Corydalis* rhizoma] (Wu et al., 2021). These are typical and widely used examples in the PCMM.

However, the processing traditional Chinese decoction is generally empirical, and there are obvious problems in the PCMM decoction pieces, such as backward or old equipment, low efficiency, no or very few digitalization and intelligence, and insufficient standardization (Yang et al., 2016; Sun et al., 2018). The development of digitalization, networking and intelligence in medicine manufacturing (Wang et al., 2020), and applying intelligent digital technologies such as intelligent sensors, real-time analysis technology and artificial intelligence to drug processing, will promote the high-quality products in TCM industry (Oztemel and Gursev, 2020).

In this review, the application of digital intelligence technology in the PCMM was reviewed on three major aspects: overview of process of PCMM, application of digital-intellectualization technology in PCMM, and digital-intellectualization industrial transformation of PCMM. The shortcomings of traditional methods in the processing of PCMM and the application significance of digital intelligence technology are shown in Table 1.

**TABLE 1:** The shortcomings of traditional processing of PCMM and the benefit of modern processing with digital intelligence technology.

Process of PCMM	The main problems in the traditional way	Digital and intelligent technologies	Significance of technology applications	References
Cleansing processing	low accuracy; low efficiency	the machine vision system; CNN; BPNN	Automatic detection and classification of medicinal materials	Shen et al. (2019); Steinbrener et al. (2019); Singh and Chaudhury (2020); Kazi and Panda (2022)
Cutting processing	Demulcent: difficult to control the process parameters during moistening	the fuzzy neural network; an intelligent vacuum gas phase displacement moistening machine	make a better control of the moistening process; maximize the retention of medicinal ingredients	Li et al. (2021); He et al. (2022)
	Cutting: facing the lost craft; empirical; significant differences in tools	deep learning architecture; computer vision technology; three-dimensional point cloud dual robot system	individualized cutting of different kinds of medicinal materials	Azarmdel et al. (2019); Chen et al. (2021); Bao et al. (2022); Liu et al. (2022); Wei et al. (2022); Yu et al. (2023)
	Drying: drying degree that cannot be controlled in real time	The delay mean rule; GA; ANN	real-time monitoring and controlling of drying	Fabani et al. (2021); Ke et al. (2022)
Stir-frying processing	outdated equipment; low degree of intelligence; empirical; fuzzy processing end point; different quality of excipients; uncontrollable heat	electronic nose; electronic tongue; cluster analysis; ANN; combining bionic sensors with chemometrics	accurately determine the end point of processing; Intelligently adjust the fire, time and temperature in the processing process	Niquet-Leridon et al. (2015); Xu et al. (2015); Huang et al. (2019); Lin et al. (2021); Zheng et al. (2022)
Quality control of decoction pieces	lack of standardization and standardization; varies in quality; low efficiency and high consumption	NIR spectroscopy combined with real-time release test and chemometrics; ANN; LS-SVM; deconvolution software; DNA metabarcoding	monitor key quality parameters of PCMM in real time; rapid qualitative and quantitative analysis of medicinal materials; accurately distinguish between genuine and counterfeit	Wang et al. (2015); Guo et al. (2019); Liu et al. (2020); Wang et al. (2020); Zuo et al. (2020); Qiu et al. (2021); Jin et al. (2022); Noroozi et al. (2022); Vecchietti et al. (2022)

## 2 Overview of process of PCMM

PCMM is a key factor affecting the quality and efficacy of decoction pieces. Usually, the botanical drugs are processed by fire after cleansing and cutting, so they are also called stir-frying. The commonly used stir-frying methods include stir-baking, steaming, boiling, and calcining. At present, in the general rules of the appendix of the 2020 edition of Pharmacopoeia of the People’s Republic of China (ChP), the processing methods are systematically, completely and scientifically classified as cleansing processing, cutting processing, stir-frying and other processing (Chinese Pharmacopoeia Commission, 2020). The processing of Chinese medicine is a dynamic process, in addition to the physical structure changes, its chemical composition may also change with different processing parameters. As mentioned above, processing PCMM is briefly divided into cleansing, cutting, stir-frying and quality control of decoction pieces. The conditions and raw materials required in different preparation methods are different, and the influencing factors are also different. Therefore, it is necessary to classify and discuss the processing.

### 2.1 Cleansing processing

Cleansing, also known as pure selection, is the first step in the processing PCMM. Generally, the purpose of removing impurities, size grading, removing non-medicinal parts and separating medicinal portions is achieved by selecting, screening, selection in wind (separating impurities from drugs by wind according to different specific gravity of drugs and impurities) and magnetic separation (using strong magnetic materials to absorb magnetic impurities in botanical drugs) to ensure the accuracy of each medicinal amount in the prescription.

### 2.2 Cutting processing

Cutting processing refers to the processing softening the purified medicinal materials with cutting them into slices, filaments, segments, blocks or other shapes with appropriate cutting tools. After cutting, it is easier to crush process, extract the effective components, and facilitate the preparation of decoctions and other preparations. In addition to fresh and dry cutting, the cutting methods may soften the medicinal materials. Generally, according to the characteristics of medicinal materials and the conditions of season and temperature, different methods such as leaching, washing, soaking, bleaching, and moistening are adopted to soften dry medicinal materials by absorbing a certain amount of moisture. The soften degree is checked by the bending method, finger pinch method, puncture method, and others in order to have a better cutting. However, at present, it is difficult to control the processing parameters during soaking and moistening. Long-term soaking may cause a significant loss of effective components. If the moistening is not appropriate, successful cutting may not be achieved (Qin et al., 2018).

The cut medicinal materials contain more water and must be dried in time to maintain the quality for decoction pieces. During the drying process, temperature, time, and drying methods affect the degree of drying, and sometimes lead to changes in metabolites or content, and biological activity (Qiao et al., 2022). The size, thickness and texture of the decoction pieces affect the drying uniformity of the decoction pieces, resulting in a large difference in the moisture content of a batch of decoction pieces. It may significantly affect the quality of the decoction medicine. However, the current moisture measurement method usually refers to the samples at a certain time interval of drying treatment, which may not actually represent the continuity of the drying process, especially during the vacuum drying process (Wang et al., 2021). Therefore, finding an online intelligent drying method to prepare decoction pieces will provide a scientific and accurate basic data for the quality control of traditional Chinese botanical drugs and the strong rational application of medicines in clinical practice.

### 2.3 Stir-frying processing

Stir-frying generally refers to a processing method of Chinese botanical drugs by fire. The heat, temperature, time, acid and alkali environment, varieties or amount of excipients, applied in the stir-frying processing affect the efficacy of processed products. It is necessary to standardize the processing, so that the effective components and botanical drugs of medicinal materials are fully retained, for ensuring the effectiveness of medicinal materials. The heat may be different with the mild fire, medium fire and martial fire. The commonly used heating methods are stir-baking, steaming, boiling and calcining. Adding different varieties or amount of excipients, the stir-frying processing may change the “four properties and five flavors” (four properties of cold, hot, warm, cool and five flavors of sour, bitter, sweet, spicy and salty) in the drug, thus may alter the therapeutic effect. Obtaining a variety of processed products of a drug may be appropriate for specific clinical treatment. Excipients are mainly divided into liquid excipients and solid excipients. The input of excipients may have an effect on the contents of botanical drugs of drugs, so as to achieve the purpose of reducing toxicity and increasing efficiency (Li et al., 2021).

Other methods include duplication, making frost-like powder, levigating, sprouting and fermentation (Chinese Pharmacopoeia Commission, 2010). *Pinellia ternata* (Thunb.) Makino [Araceae; *Pinelliae* rhizoma], *Arisaema erubescens* (Wall.) Schott [Araceae; *Arisaematis* rhizoma], *Sauromatum giganteum* (Engl.) Cusimano and Hett. [Araceae; *Typhonii* rhizoma] and other Araceae botanical drugs are often processed by duplication method.

Fermentation method requires controlled environment of temperature, humidity, moisture, *etc.*, and the fermentation processing must be completed in one processing step without interruption. The temperature and humidity, pH value and medium substrate should be checked and monitored in real time to avoid unwanted bacterial growing, mildew and rancidity caused by environmental factors. In addition, making frost-like powder, levigating and dry distillation are also the characteristics of PCMM. In these processing methods, firepower, temperature, time, water addition and excipients may also affect the quality of decoction pieces to a certain degree.

### 2.4 The main problems of quality control of decoction pieces

The processing is a key factor to ensure the safety and effectiveness of Chinese botanical drugs. At present, the PCMM has a good science and technology foundation and careful scale measurement. There are, however, still some difficulties, such as the characteristic processing technology on the verge of being lost, the lack of innovation, the lack of brand quality, and the lack of precise clinical application, *etc.* The quality of Chinese medicine decoction piece is still difficult to quantify, to evaluate, to control, and to maintain efficiency, through the extensive processing steps. These difficulties become the key scientific limitation restricting the development of Chinese botanical drugs industry.

## 3 Applications of digital and intelligent technologies in process of PCMM

Digitization is the basic process of converting many complex and changeable information into measurable numbers, and then establishing appropriate digital models based on these numbers, transforming them into a series of binary codes, and introducing them into the computer for unified digital processing. Intelligence refers to the attributes of things that may meet various needs under the support of computer network, big data, internet, and artificial intelligence. The initial definition of digital and intelligent technologies is ***the combination of digital intelligence and intelligent digitalization***. At present, the main branches of artificial intelligence applications include machine learning (ML), machine vision, Internet of Things (IoT), artificial neural network (ANN), robotics and expert system, *etc.* (Kutyauripo et al., 2023). Digital technology, artificial intelligence and other technologies continue to grow, and digital intelligent technology has been widely used in the daily life. For example, self-driving cars use the most reasonable autonomous driving scheme according to the information of the surrounding scene, identified driving intention, and intelligently responsive emergencies (Shi et al., 2022).

Due to the objectivity and intelligence of digital intelligence technology, researchers in the medical field use them to automatically detect and identify organ dysfunction to optimize disease diagnosis and treatment (Ehrenreich et al., 2020; Hatanaka, 2020). As an important part of improving clinical efficacy, the PCMM processing is a cumbersome, complex, diversed and influencing factor. Moreover, the whole processing is decided by pharmacists based on experience, which may have obvious limitations. The lack of accurate processing quality control technology is likely to greatly impact the efficacy of drug. At the same time, due to the complex chemical reactions along the processing, the unforeseeable degree of effective variation, the lack of reliable online instruments, the complex modeling, the lack of visualization technology, the lack of efficiency, the low sensitive detection, and the lack of real-time monitoring technology, the most optimized processing is still hard to achieve. Therefore, it is necessary to introduce digital intelligence technology into the processing control of traditional Chinese medicine using objective quantitative indicators to accurately regulate the processing steps.

### 3.1 The application of digital intelligence technology in cleansing processing

The machine vision system may quickly obtain a large amount of information, which is more efficient and accurate than manual selection. It is easy to process automatically and integrate with designed and processing-controlled information; which is highly suitable for modern automated production processes. Convolutional neural networks (CNN) has powerful visual information on processing capabilities and may achieve efficient hierarchical feature expression of data. CNN plays an important role in applications such as image classification and detection in processing control (Kazi and Panda, 2022). Therefore, based on the CNN recognition method, the data sets of different medicament portions and various impurities may be constructed. The Wiener filtering algorithm and multi-scale enhancement algorithm are used for image preprocessing to identify different medicinal parts and impurities (Shen et al., 2019; Steinbrener et al., 2019). The local texture feature extraction technology is used to form texture features. The performance of the proposed feature set is compared with the existing techniques based on running length matrix, co-occurrence matrix, size region matrix, neighborhood gray tone difference matrix and wavelet decomposition. The backpropagation neural network (BPNN) may also classify the same medicinal materials of different origin and species (Singh and Chaudhury, 2020). In addition to screening the image features of medicinal materials, it may also be combined with various sensors to quantify the shape, color, taste and other indicators of Chinese medicinal materials, so as to select impurities, non-medicinal parts and different medicinal portions respectively (Yin et al., 2021; Wang et al., 2022).

### 3.2 The application of digital intelligence technology in cutting processing

In the process of softening, many water-soluble active ingredients may be lost or destroyed to varying degrees. Li et al. (2021) developed an intelligent vacuum gas phase displacement moistening machine. By controlling the vacuum degree and infiltration time, the medicinal materials were softened quickly and evenly with low water content, and reduced loss of active ingredients during the process of infiltration. At the same time, this improved vacuum played an important role in reducing the energy consumption of subsequent drying, improving work efficiency, and reducing production costs. He et al. (2022) designed a predictive control method of the processing temperature of Chinese medicine decoction pieces based on the fuzzy neural network, in order to solve the problem that the temperature of Chinese medicine decoction pieces was difficult to control during gas phase replacement wetting. This method realized the optimal automatic control of the temperature tracking set value of Chinese medicine decoction pieces in the processing step of gas phase replacement wetting. All these new intelligent technologies make a better control of the moistening processing of Chinese medicine decoction pieces, maximize the retention of medicinal ingredients, and improve clinical efficacy of the drugs.

As for cutting, different kinds of medicinal materials may be discussed separately. Combining advanced computer vision technology with deep learning architecture, the image data sets of different kinds of plant medicinal materials are constructed. The mathematical model of rhizome cutting specification is established; and the cutting of different specifications and tablets is designed by intelligent conversion of blade position (Yu et al., 2023). The circular mask large template was used to detect the seed morphology, the data distribution was used to locate the spindle and distinguish the different parts of the seed to obtain the slices of the seed medicinal materials (Wei et al., 2022). Through the cutting and coring mechanism of flower medicinal materials, the cutting and coring processes at different positions are realized to obtain flower medicinal materials such as pollen, stigma and inflorescence (Chen et al., 2021). Animal medicinal materials may be analyzed by machine vision and three-dimensional point cloud dual robot system, using 3D scanning system, corpse fixing device and laser-guided efficient cutting robot, to develop a system that may intelligently grab, clean and cut various animal tissues of different sizes and shapes (Azarmdel et al., 2019; Bao et al., 2022; Liu et al., 2022). The delay-mean-rule is designed by simulated annealing algorithm and time window variable optimization method. Such rule is used for real-time monitoring and controlling of drying of medicinal materials by dryer equipment (Ke et al., 2022). The genetic algorithm (GA) was used to optimize the ANN to simulate the drying processing, the best drying curves was obtained, and green drying method was selected (Fabani et al., 2021).

### 3.3 The application of digital intelligence technology in stir-frying processing

In the PCMM, heat refers to the size and duration of fire when the drug is heated, which is a key factor affecting the quality of decoction pieces. Studies have shown that with the change of fire degree, the content of volatile components, amino acids and reduced sugars in drugs have changed significantly (Chen et al., 2016; Hu et al., 2016). Therefore, through the digital technology, the various influencing factors in the processing are objectively quantified, and then the artificial intelligence technology is used to adjust and control the fire, time, temperature and processing end point, which is conducive to the standardization of processing technology and the quality of decoction pieces. As such, it realizes the intelligent and modern production of decoction pieces.

The contents of chemical components and biological activity of drugs may be changed by steaming, boiling, and frying under different air pressures (Trakoontivakorn et al., 2012; Guo et al., 2022). Maillard reaction often occurs in the heat treatment process of food and pharmaceutical, which is the main chemical reaction to produce new flavor and new color in the processing food and Chinese botanical drugs. With the increase in temperature, Maillard reaction often shows an increasing trend too (Gerrard, 2006; Zhang, 2020). Xu et al. (2015) compared the raw betel nut with its processed products of stir-frying yellow, stir-frying coke and stir-frying charcoal, and found that the contents of 5-HMF and arecoline in the three processed products were significantly different and closely related to the levels obtained by electronic nose and electronic tongue. Zheng et al. (2022) used cluster analysis and Artificial neural network analysis (ANN) to better identify gardenia with various processing degrees. For the determination of the end point of processing, the color difference and distribution in the image may be used to determine the degree of stir-frying and cooking of the drug, so that the processing system may determine whether to use partial tumbling, or to end the processing when the decoction pieces reach the optimal efficacy; such processing system may avoid improper or excessive processing (Lin et al., 2021). By combining bionic sensors with chemometrics, the overall quality evaluation system of decoction pieces was established and used to determine whether the processing time of decoction pieces reached sufficient with the most optimized quality (Zhang et al., 2023). In addition, fermentation drugs were processed within antibacterial biomolecules-covalent organic frameworks through a combination of artificial intelligence technologies, such as low-cost sensors, machine learning (ML), and intelligently release antibacterial substances according to pH values, to intelligently regulate and evaluate the drug fermentation processing (Viejo and Fuentes, 2020; Qiao et al., 2021).

### 3.4 Application of digital intelligence technology in quality control of decoction pieces

Due to the particularity of Chinese medicine decoction pieces, it is not only necessary to control the process of PCMM but also to test the quality of the final pieces to ensure the safety and effectiveness of the drug. At present, many scholars have achieved stable and rapid qualitative and quantitative analysis through new technical methods such as electronic sensors, Fourier transform near-infrared technology (Lan et al., 2020), Desorption electrospray Ionization mass spectrometry (DESI-MSI) combined with metabolomics (Liu et al., 2022), Static headspace-multi-capillary column with gas chromatography coupled to ion mobility spectrometry (Cao et al., 2014) and Process Analysis Technique (PAT) based on chemometrics (Korasa and Vrečer, 2018; Kamyar et al., 2021). Near Infrared Spectrum (NIR) spectroscopy has become the most commonly used PAT process analyzer in pharmaceutical technology because of its advantages of non-destructive measurement and real-time monitoring in the process, and is particularly suitable for complex production that requires process quality control (Lee et al., 2019). Therefore, it is urgent to find technical methods that may monitor key quality parameters in real time in the processing, and obtain high quality slices through continuous monitoring the process, so as to eliminate the unqualified final quality of slices that may be caused by improper processing.

Jin et al. (2022) and Zuo et al. (2020) monitored, in real-time, the online quality of industrial concentration processing of *Lonicera japonica* Thunb. [Caprifoliaceae; *Lonicerae japonicae* flos] and the steaming processing of *Gastrodia elata* Blume [Orchidaceae; *Gastrodiae* rhizoma] by NIR spectroscopy as a processing analysis tool combined with real-time release sensor and chemometrics. Thus, the quality of drugs and therapeutic effect were significantly improved. Qiu et al. (2021) reported that enzymatic browning was the main factor of metabolic changes during the processing of *Salvia miltiorrhiza* Bunge [Lamiaceae; *Salviae miltiorrhizae* radix et rhizoma] using metabolomic data, which was helpful to select the best processing technology of *Salviae miltiorrhizae* radix et rhizoma according to the actual needs. Therefore, a combination of NIR spectroscopy, metabolomics, Gas Chromatography-ion Migration Spectrometry (GC-IMS) (Xu et al., 2022), online stepwise background subtraction-based Ultra high performance Liquid chromatography-Quadrupole-time-of-Flight Tandem Mass Spectrometry (UHPLC-Q-TOF-MS/MS) dynamic detection (Guo et al., 2022) and other digital intelligence techniques should be considered to monitor the odor, metabolic components and chemical components during the PCMM processing, and to provide real-time and data-based test results to the cloud, with reduced falsification of testing and optimized drug quality. The content of active ingredients in Chinese medicinal materials, intermediate products and processed products may be rapidly determined by ANN and Least squares support vector machine (LS-SVM) combined with spectrophotometry or Fourier-Transform near infrared (FT-NIR). The deconvolution software may be used to qualitatively and quantitatively analyze the impurities of medicinal materials by HPLC and HPLC-Photodiode array detector (PDA) systems (Wang et al., 2020; Qiu et al., 2021; Noroozi et al., 2022; Vecchietti et al., 2022). For some new chemical components of processed products, the influence of their chemical characteristics on performance is used to predict their effects in the patients through machine learning (ML) models to prevent drug-induced damage (Mcloughlin et al., 2021). DNA barcoding may quickly detect the quality of Chinese medicine decoction pieces at the molecular level, which is efficient, reliable, sensitive and repeatable, and may ensure the safety of clinical medication (Wang et al., 2015; Xin et al., 2018). In addition, for some valuable medicinal materials that are easily adulterated, UHPLC-Q-TOF-MS, PCA methods may be combined with Progenesis QI, Makerlynx XS and other software to identify medicinal materials according to specific characteristic markers, and to accurately distinguish between genuine and counterfeit (Guo et al., 2019; Liu et al., 2020).

## 4 Digital intelligent research and industrial transformation of PCMM

Örs et al. (2020) proposed operational digital twin (DT) for chemical processes, aiming at data management, processing detection, processing modeling, processing optimization, production scheduling, and advanced processing control. A system based on DT was proposed to regulate the processing. Sensors were used to collect data, and the medicinal materials were identified and classified by machine vision, smell and CNN. Personalized or optimized processing was carried out according to the characteristics of each batch of medicinal materials. At the same time, a system designed by the concept, may analyze and control the processing through real-time measurements to ensure the processing moderation and the quality of final decoction pieces, so as to transform the PCMM to the digital intelligence environment. Through the intelligent control of each step of the processing, each processing is fully executed throughout every step, with strictly controlled quality of decoction pieces, carefully monitored digital characterization of PCMM, and using novel intelligent control of traditional Chinese medicine processing. This section will progress from the research and development of TCM processing laboratory and the processing line of factory, to the digital intelligence technology not only to the processing trial, but also to the industrial production lines.

### 4.1 Lab test

Through data mining (DM), the database of raw and processed products of traditional Chinese medicinal materials was established; the internal relationship between raw and processed products of traditional Chinese medicine and processing steps was analyzed; the related model of processing and quality of decoction pieces was constructed; and the conditions and regulation of processing steps were established (Fusheng et al., 2009). Based on the model of the efficacy of traditional Chinese medicine formula (Liu et al., 2022), a weighted calculation method related to the processing conditions such as temperature, types and methods of excipients, may be constructed. The graph convolutional network (GCN) is combined with the conditions in the processing steps to predict the efficacy of processed products.

The research on the technology in producing PCMM is an important link to realize the standardization of the traditional Chinese medicine processing. ML is the core of artificial intelligence, which may predict new and invisible data based on previous observed results and data, so that the computer may automatically learn without manual intervention, to adjust the operation accordingly, and to make decisions automatically. It is often divided into three categories: supervised learning, unsupervised learning and reinforcement learning (Kersting, 2018; Lins and Givigi, 2021). The lab test may discover the changing components during the processing by CNN and deep learning (DL), so that the quality markers of decoction pieces, standardizing the PCMM technologies, controlling the quality of decoction pieces, and formulating the standard of decoction pieces may be achieved (Skrede et al., 2020). In addition, the fuzzy logic method may be used to explore the effects of different processing methods on the sensory properties of decoction pieces without reducing the efficacy, and appropriate processing methods may be selected to improve the appearance and taste of decoction pieces (Sasikumar et al., 2019; Deba-Rementeria et al., 2023).

### 4.2 Intelligent industrial production of traditional Chinese medicine decoction pieces

With the advent of the fourth industrial revolution, the future factory will be highly digital and intelligent to meet the needs of real-time, flexible, personalized and automated monitoring of traditional Chinese medicine processing, and will realize the intelligent production of ‘Internet + manufacturing’ (Zunino et al., 2020). The online detection technology, Internet digital technology and artificial intelligence control technology are running through the automatic processing equipment and production line. The computer information management system of drug processing and the digital intelligent production line of Chinese medicine decoction pieces can be established (Shi et al., 2022). The establishment of a standardized system of processing equipment and processing parameters is the key factor to promote the modernization of Chinese medicine decoction pieces. The combination of digital intelligence technology and the equipment system of purification, cutting and processing, including washing machine, screening machine, cutting machine, stir-frying machine, and boiling pot, gradually establish a production line of Chinese medicine processing equipment in line with the characteristics of produced Chinese medicine with appropriate integration and automation. Follow the ancient processing technology, combined with modern digital intelligence technology and new intelligent processing equipment, a production line with high efficiency, energy saving, continuous production and controllable quality may be created to improve the manufacturing level of Chinese botanical drugs industry, and to accelerate the transformation of traditional Chinese decoction pieces industry to digital intelligence (Shi et al., 2022; Zhu et al., 2022).

#### 4.2.1 Digital and intelligent equipment of PCMM

Hardware equipment is the infrastructure of the industrial production. With the acceleration of the modernization of TCM, the processing equipment needs to be constantly innovated. The standardization of traditional Chinese medicine processing equipment is the premise of the standardization of processing parameters. The development of digital intelligent processing equipment is helpful to solve the modernization problem of TCM decoction piece industry.

The sensor can objectively show the shape, color and taste of the drug through the digital form of the sensitive element, which is the first link to realize automatic detection and control. New sensors are added to the processing equipment, and the corresponding mathematical model is established. The artificial intelligence system is used for automatic control, and the technological innovation is achieved to promote the new equipment creation. The traditional processing technologies such as levigating, dry distillation, duplication, and roasting are combined with modern microwave technology, cyclone and foam separation, mass transfer and heat transfer, and biotransformation to develop new processing equipment.

The stir-frying equipment is the most characteristic pharmaceutical equipment of traditional Chinese medicine. Taking the stir-baking equipment as an example, the stir-frying machine is transformed into a fuel gas heat source supply and automatic temperature control system, which may not only achieve rapid heating, but also use the real-time, continuous and non-contact measurement of the temperature in the pot by a temperature sensor. It is truly the digital monitored and managed PCMM processing (Yang et al., 2022). Non-contact infrared temperature measurement uses infrared thermal imaging technology and ANN-based data-driven algorithm to estimate the temperature of medicinal materials. This system realizes dynamic continuous non-contact temperature measurement, and monitors the temperature parameters during processing (Liu et al., 2022). The online detection system of traditional Chinese medicine processing equipment combines the intelligent sensory technology and online monitoring technology to digitally express the processing steps and realize the objectification of the PCMM. The application of internet, internet of things (IoT), big data and computer technology improves the traditional Chinese medicine processing equipment, promotes the informatization and automation of processing, optimizes the quality of decoction pieces, accelerates the transformation, and upgrades the traditional Chinese medicine decoction pieces industry.

#### 4.2.2 Digital and intelligent production line

**FIGURE 1:**
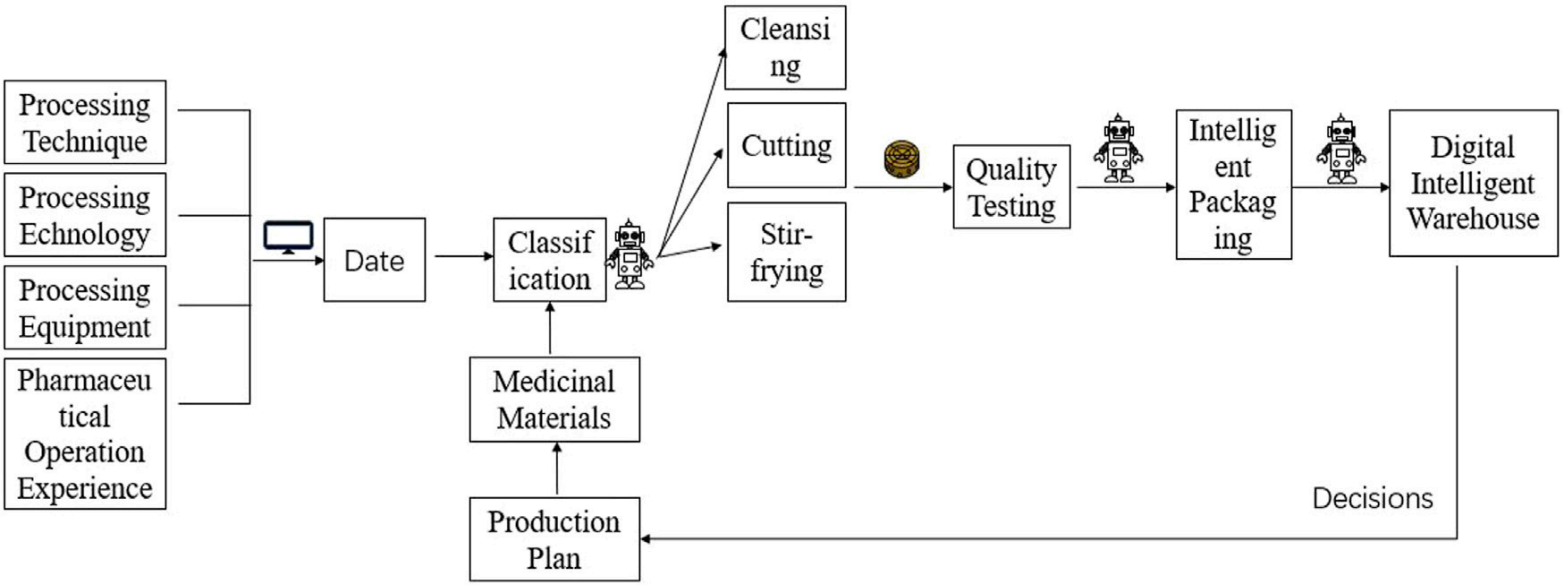
Schematic diagram of digital intelligent production line of Chinese decoction pieces.

Based on the application of the above-mentioned digital intelligent technology in the processing and the digital intelligent processing equipment, an efficient digital intelligent production line of Chinese medicine decoction pieces may be designed (Figure 1). By automating the processing of large quantities of medicinal materials while making the processing digital and intelligent, high-quality, and effective decoction pieces may be produced. Digital intelligent processing factory may use the IoT technology combined with the other advanced technologies, such as industrial big data, Wireless Sensor network (WSN), Radio Frequency Identification (RFID), and cloud computing platforms, to achieve intelligent management by monitoring the use of materials in the production line and maximizing the efficiency of optimized production (Liu et al., 2020). This digital intelligent processing factory may integrate all medicinal materials, processing techniques, processing equipment and pharmaceutical operations into digital data through big data, deep learning, case analysis and simulation modeling technologies. Through RGB image data, convolutional neural network and discriminant function, each medicinal material is classified, and transported to different processing workshops by transportation robots. Appropriate processing is carried out according to the nature of the drug itself and the medicinal needs. The corresponding mathematical model is established to ensure that the key processing parameters are optimized to maintain a high product quality (Mears et al., 2017). The decoction pieces produced in each processing workshop are sent to the laboratory for rapid, accurate and non-destructive quality testing. The qualified decoction pieces are classified and packaged with suitable multifunctional biodegradable intelligent packaging materials. The processing steps may be traced through the intelligent functions of microbial spoilage biosensor, detection, recording, tracing and communication (He and Shi, 2021; Khan et al., 2023; Kong et al., 2023; Palazzo et al., 2023). Subsequently, the decoction pieces are transported to a number of intelligent warehouses with different storage conditions. The system analyzes the quantity of ordered products according to the collection, monitors the real-time inventory in the warehouse, intelligently adjusts the processing variety and quantity, rationally saves the storage space in the warehouse, and reduces the risk and cost of expired products (Ahmadi et al., 2022). Through integrated software such as cloud computing and big data, with automatic data collection and decision-making, dynamical regulation between departments may be achieved (Coito et al., 2020).

**FIGURE 2:**
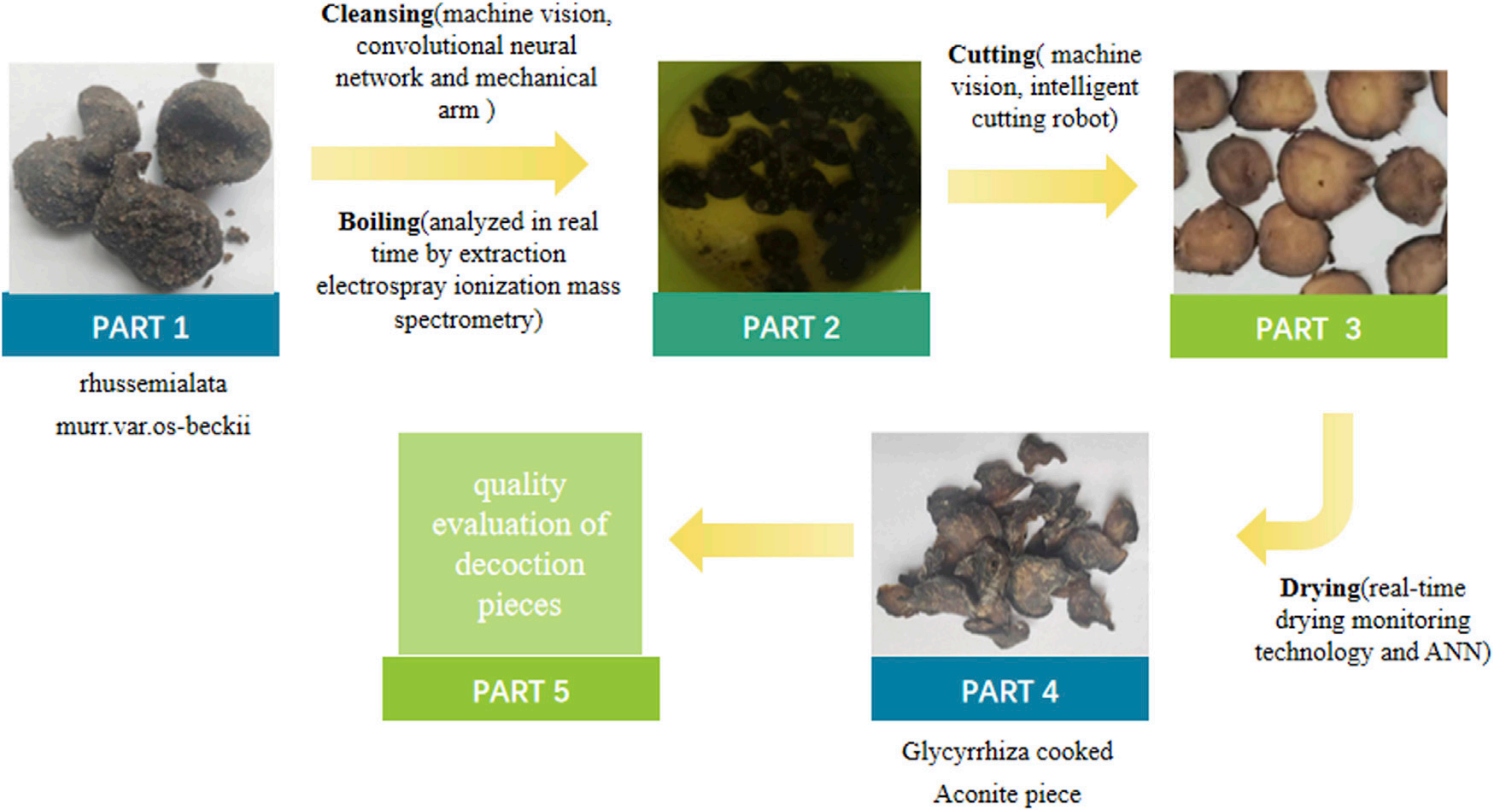
Processing flow chart of aconite.

Take Fuzi (*Aconite lateralis* radix praeparata), an important medicine for restoring “Yang” and rescuing patient from collapse, as an example (Figure 2). Aconitum carmichaeli contains a large number of diester alkaloids, which have strong toxicity. The processed products from it are generally used in clinic with reduced toxicity, and there are many kinds of processed products (He et al., 2023). Herbon mass spectrometry, and the appropriate fitting function was selected intelligently to reduce the toxicity of aconite (Qiu et al., 2020). Finally, machine vision, intelligent cutting robot, and real-time drying monitoring technology were used to cut aconite into appropriate thickness and dried for clinic use.

#### 4.2.3 Other auxiliary systems

In addition to the equipment and technology required for processing, the digital intelligent processing factory also needs to detect faults and abnormalities in real time, and applies the intelligent alarm system to provide detection and diagnosis of abnormal events and solutions to minimize emergency closure during processing and to maintain efficient and continuous production (Gupta et al., 2013; Velasquez et al., 2022). Under the digital monitoring and intelligent control, the actual production processing is constantly monitored and right decision is rapidly achieved to maintain the production line at the most optimal level. This result promotes the intelligent control of the workshop, creates a machine automation workflow, and pursues robust and adaptive decision in the workflow through artificial intelligence thinking and learning (Fu et al., 2019; Ng et al., 2021). By using the auxiliary technology of brain-computer interface to enhance and develop novel technology, by sensing the temperature and imagine of the environment, and by monitoring the water use of the factory to intelligently control the lighting, temperature and water valve switch of the factory, the factory is running at the most optimized condition with saved resources (Al-Hudhud et al., 2019; Uysal and Mergen, 2021). The intelligent system is developed to detect the content of drugs in the sewage of the processing plant, to predict the drug contents and the influence by re-irrigated plants after treatment, to reasonably control the drug content in the sewage, and to realize the green and sustainable production mode (González García et al., 2019).

## 5 Conclusion

Chinese medicine processing is very important to the preparation and use of Chinese medicine. Many countries in the world have taken the green and sustainable development as an important direction of scientific and technological revolution and industrial transformation. The new generation of information technology is rapidly progressing and the digital economy is expanding in scale. In this context, grasping the opportunity of technological iteration, accelerating the digitalization and intelligent transformation, and upgrading of the TCM industry have become an important starting point to promote the high-quality sustainable development of the TCM industry. Digital intelligent transformation is the combination of digital transformation and intelligent elements (such as artificial intelligence, Internet of Things and other technologies). Numeral intelligent processing makes all traditional experience objective and information, production processing and quality control, into digital and intelligent controlled processing. New technology aims to improve the problems of distinguishing, evaluating and controlling the quality of traditional Chinese medicine decoction pieces through extensive processing with low efficiency. Through the multi-dimensional holographic processing property characterization, the electronic rapid identification of the shape and taste quality of decoction pieces, and the intelligent control of fire temperature and other technologies, the traditional processing technology may be thoroughly innovated and improved. The scientific content of TCM may be improved. The modern development needs may be met. The clinical application of characteristic decoction pieces may be expanded. The high-quality industrial benefits may be achieved. The intelligent manufacturing of a number of traditional Chinese medicine decoction pieces may be achieved. This paper reviewed the application of digital intellectualization technology in the processing of TCM, which promoted the standardization of the processing, the standardization of the quality of decoction pieces, the digitalization and intellectualization of production, and the safety and effectiveness of clinical use of TCM decoction pieces. At the same time, this review also provided a theoretical basis for the technical progress and high-quality development of TCM industry in the future.

## Abbreviations

ANN, artificial neural network; BPNN, backpropagation neural network; ChP, Pharmacopoeia of the People’s Republic of China; CNN, convolutional neural networks; DESI-MSI, desorption electrospray ionization mass spectrometry Imaging; DM, data mining; DSS, decision support system; DT, digital twins; FT-NIR, fourier-transform near infrared; GA, genetic algorithm; GC-IMS, gas chromatography-ion migration spectrometry; GCN, Graph Convolutional Networks; 5-HMF, 5-hydroxymethylfurfural; IoT, internet of things; LS-SVM, least squares support vector machine; MIR, middle infrared; ML, machine learning; NIR, near infrared; PAT, process analysis technology; PCA, principal component analysis; PCMM, Processing of Chinese Materia Medica; PDA, photo-diode array; PLS-DA, partial least squares discriminant analysis; RFID, radio frequency identification; RTRT, real-time release test; TCM, traditional Chinese medicine; UHPLC-Q-TOF-MS/MS, ultra-high pressure liquid chromatography quadrupole time of flight tandem mass spectrometry; WSN, wireless sensor networks.
